# Oxidative Stress and Analysis of Selected SNPs of *ACHE* (rs 2571598), *BCHE* (rs 3495), *CAT* (rs 7943316), *SIRT1* (rs 10823108), *GSTP1* (rs 1695), and Gene *GSTM1*, *GSTT1* in Chronic Organophosphates Exposed Groups from Cameroon and Pakistan

**DOI:** 10.3390/ijms21176432

**Published:** 2020-09-03

**Authors:** Leonel Javeres Mbah Ntepe, Rabia Habib, Ngondi Judith Laure, Saqlain Raza, Eugenie Nepovimova, Kamil Kuca, Sajida Batool, Syed Muhammad Nurulain

**Affiliations:** 1 Department of Biosciences, COMSATS University Islamabad, Chak Shahzad, Islamabad 45550, Pakistan; mbahjl@yahoo.fr (L.J.M.N.); sajida.batool@comsats.edu.pk (S.B.); 2 Department of Biochemistry, Yaoundé I University, Yaoundé 8024, Cameroon; ngondijudithl@hotmail.com; 3 Department of Mathematics, COMSATS University Islamabad, Chak Shahzad, Islamabad 45550, Pakistan; saqlain.raza@comsats.edu.pk; 4 Department of Chemistry, Faculty of Science, University of Hradec Kralove, 50003 Hradec Kralove, Czech Republic; eugenie.nepovimova@uhk.cz

**Keywords:** organophosphates, antioxidants, cholinergic enzymes, SNPs, toxicogenetics

## Abstract

The detrimental effects of organophosphates (OPs) on human health are thought to be of systemic, i.e., irreversible inhibition of acetylcholinesterase (AChE) at nerve synapses. However, several studies have shown that AChE inhibition alone cannot explain all the toxicological manifestations in prolonged exposure to OPs. The present study aimed to assess the status of antioxidants malondialdehyde (MDA), superoxide dismutase (SOD), glutathione (GSH) (reduced), catalase, and ferric reducing antioxidant power (FRAP) in chronic OP-exposed groups from Cameroon and Pakistan. Molecular analysis of genetic polymorphisms (SNPs) of glutathione transferases (*GSTM1, GSTP1, GSTT1*), catalase gene (*CAT*, rs7943316), sirtuin 1 gene (*SIRT1*, rs10823108), acetylcholinesterase gene (*ACHE*, rs2571598), and butyrylcholinesterase gene (*BCHE*, rs3495) were screened in the OP-exposed individuals to find the possible causative association with oxidative stress and toxicity. Cholinesterase and antioxidant activities were measured by colorimetric methods using a spectrophotometer. Salting-out method was employed for DNA extraction from blood followed by restriction fragment length polymorphism (RFLP) for molecular analysis. Cholinergic enzymes were significantly decreased in OP-exposed groups. Catalase and SOD were decreased and MDA and FRAP were increased in OP-exposed groups compared to unexposed groups in both groups. GSH was decreased only in Pakistani OPs-exposed group. Molecular analysis of ACHE, BCHE, Catalase, GSTP1, and GSTM1 SNPs revealed a tentative association with their phenotypic expression that is level of antioxidant and cholinergic enzymes. The study concludes that chronic OPs exposure induces oxidative stress which is associated with the related SNP polymorphism. The toxicogenetics of understudied SNPs were examined for the first time to our understanding. The findings may lead to a newer area of investigation on OPs induced health issues and toxicogenetics.

## 1. Introduction

Pesticides are an important class of environmental chemical pollutants. Their steady and continuous use for more than half a century has now become a major public health problem, killing at least 250,000–370,000 people every year [1]. Pesticides exposure is one of the notable contributors to morbidity particularly in the developing part of the world [2]. Epidemiological studies have found that pesticide exposure may result in metabolic disorders, infertility, neurological problems, or weak immune system. Insecticides have also been linked to human DNA damage, multiple degenerative diseases, and cancer [3,4,5]. Organophosphates (OPs) are one of the widely used pesticides (more than 50% of world consumption) globally and their use is expected to increase even more in coming years, with the direct consequence of increased health problems [6,7]. Adverse health effects of OPs have been attributed predominantly to their systemic actions, i.e., they block the degradation of acetylcholine at cholinergic synapses by irreversible inhibition of acetylcholinesterase (AChE) and butyrylcholinesterase (BChE) [8,9]. However, several studies have revealed that inhibition of cholinesterase alone is insufficient to explain the varied range of disorders reported after OPs exposure [10,11,12]. Consequently, a search of other associated mechanisms to explain the toxicity of OPs was deemed necessary. Several toxicological and epidemiological studies thus far have shown that OPs can induce oxidative stress (OS) by increasing reactive oxygen species (ROS) and reactive nitrogen species (RNS). These reactive oxygen and nitrogen intermediates can react with biological macromolecules altering their physiological functions [13,14,15]. For instance, the free radicals (FR) can induce depolarization of the mitochondrial membrane, accompanied by generation of ROS and release of numerous pro-apoptotic proteins, such as cytochrome C, which can lead to cell death by apoptosis [16,17,18]. FR can also cause genotoxicity by lipid peroxidation which leads to chromosomal breaks, single-stranded DNA breaks with serious consequences on replication or transmission of a genetic message and proteins synthesis [19,20].

Antioxidant enzymes like the superoxide dismutase (SOD), glutathione peroxidase, malondialdehyde (MDA), catalase (CAT), and glutathione (GSH) act as endogenous free-radical scavengers to limit the damage caused by OS. However, pesticides can interfere with these enzymatic activities damaging antioxidant defenses, thus affecting their ability to fight oxidative stress. Many studies have explored the relationship between pesticide exposure and OPs with varying and controversial results. Those studies have assessed various tissues and blood at diverse doses and exposure surroundings (either acute or chronic). Prakasam et al. and Rastogi et al. [21,22] show a marked surge in MDA in those exposed to organophosphate pesticides. The rise in CAT and SOD have also been described by different studies [23,24,25]. On the other hand, Lu et al. [26], showed a decrease in SOD, CAT, and GSH in OP-exposed individuals. Ahmad et al. [27] showed that GST protects lungs and brain from toxic effects of pesticides. One major challenge in work-related pesticide exposure exploration stems from the unevenness in individual reactions and genetic susceptibilities, and variances in sensibilities to a particular chemical. Genetic polymorphisms (SNPs) of the pesticide-metabolizing enzymes may also affect the toxicity of pesticides and boost or reduce the sensitivity to some of the compounds. It has been reported that subjects with null genotypes for GSTM1 and GSTT1 are more pesticides susceptible [28]. These reports clearly indicate that oxidative stress and associated SNPs have an influence in the pathogenesis-related to OPs pesticide toxicity. However, only few studies have investigated the effects of chronic OPs exposure on human health so far.

This study was therefore undertaken to establish if continued low-level exposure to mixtures of organophosphorus pesticides could result in alterations in antioxidants and cholinergic enzymes. In addition, impact of SNPs of some antioxidants and cholinergic enzymes; glutathione transferases (GSTM1, GSTP1, GSTT1), *CAT* (rs7943316), *ACHE* (rs2571598), *BCHE* (rs3495), and *SIRT1* (rs10823108) has been evaluated for the first time to our understanding. The study mainly evaluated the interactions between OPs exposure and genetic polymorphisms and their impact on antioxidants enzymes activities. Only a few studies have been conducted on gene-environment interactions in populations exposed to organophosphorus pesticides in general and no such data is available for Central Africa. Beyond the process of early diagnosis, prevention and non-OP-exposed of diseases associated with long-term exposure to Ops. Our study will provide the crucial evidence on the need for individualized health interventions or pharmaco-genetics based therapeutic approaches.

## 2. Result

### 2.1. Sociodemographic and Clinical Profile of Subjects Exposed to Ops

The distribution of the population according to socio-demographic characteristics (Table 1) shows that majority of exposed Pakistanis and Cameroonians were between 16 and 30 years old. Sex ratio (Male/Female) was 2.85 in the Pakistani group against 1.00 in the Cameroonian group. 44.5% of Pakistani population were overweight (BMI ≥ 25), 25% were obese, 56% had high systolic blood pressure (B.P.), and 56.5% had high diastolic blood pressure. In the exposed population of Cameroon, 45.5% were overweight, 34% were obese, 58.5% had high systolic, and 66% had high diastolic blood pressure.

### 2.2. Pesticide Exposure and Effects on Oxidative Stress

Cholinergic enzyme activities are shown in Table 2. 44.42% Pakistani and 42.86% RBC-AChE in Cameroonians exposed individuals were depressed in comparison to non-exposed non-OP-exposed group. Reduction in activity is statistically significant. BChE activity was also decreased (Table 2) and statistically significant (*p* < 0.05).

**Figure 1:**
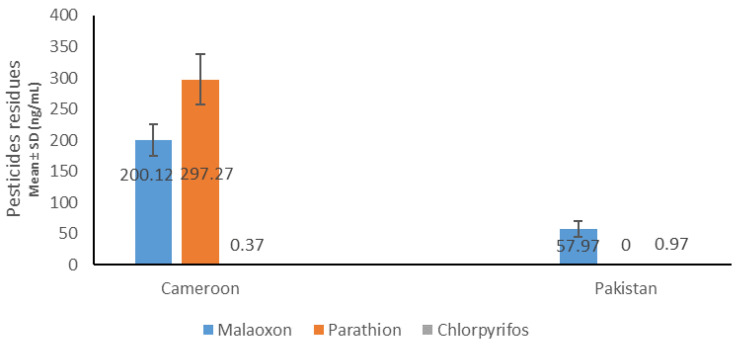
Blood pesticides levels in Cameroonian and Pakistani groups.

OPs residues determination by GC–MS (Figure 1) revealed the presence of three Ops: malathion, (Mean ± SD: 200.12 ng/mL ± 25.14), parathion (Mean ± SD: 297.27 ng/mL ± 40.29), and chlorpyrifos (Mean ± SD: 0.37 ng/mL ± 0.09) in the Cameroonian OP-exposed group. No parathion residue was found in the Pakistani OP-exposed group in our detection range. Malathion was (57.97 ng/mL ± 12.73) and chlorpyrifos was found to be (0.97 ng/mL ± 0.07) present in Pakistani group. For antioxidant enzymes (Table 3), a reduction in catalase, SOD, GSH (reduced), and rise in MDA and FRAP were observed in exposed subjects which were significant (*p* ≤ 0.05) except GSH in Cameroonian population.

### 2.3. Genetic Association Analysis

Allele and genotype frequencies results of all SNPs are summarized in Table 4. For GSTP1 rs1695, statistically significant differenes was noted in the genotypes frequencies in Cameroonian OP-exposed groups compared to healthy controls. Out of 200 exposed subjects, 42% were homozygous for the major allele, 37.5% were heterozygous GA, while 20.5% were homozygous for G minor allele. Significant association of GSTP1 rs1695 SNP with effects of OPs exposure in Cameroonian OP-exposed individuals was detected in DM and allelic models.

**Figure 2:**
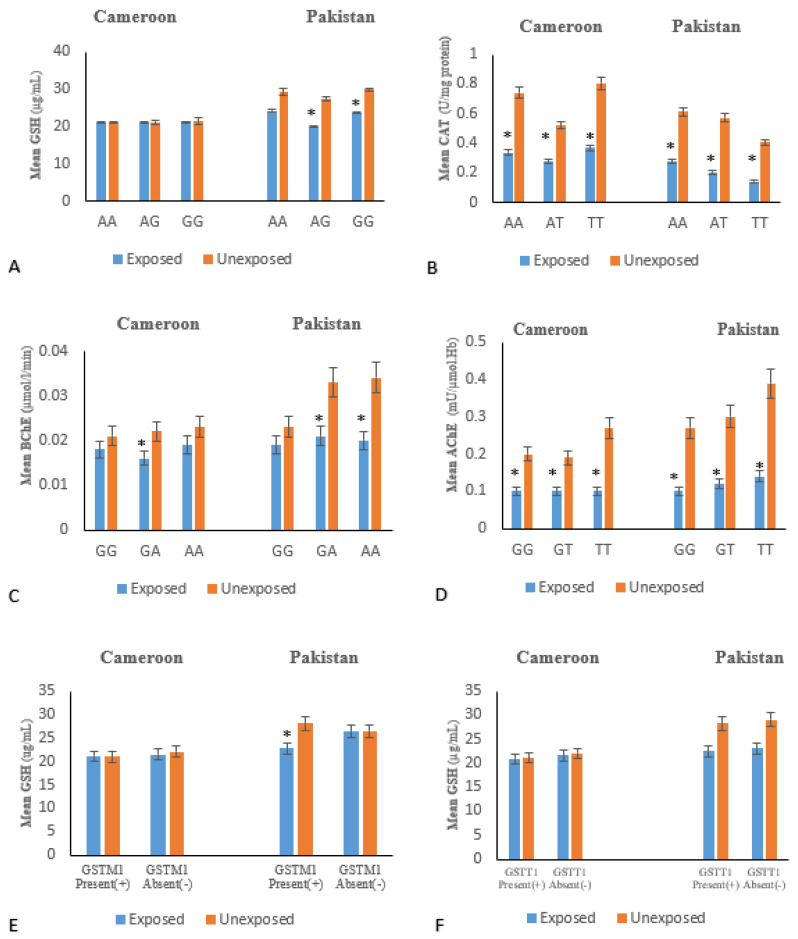
Variation of biochemical parameters according to different genotype: The results were significant if * *p* ≤ 0.05. (**A**) *GSTP1* gene; (**B**) *CAT* gene; (**C**) *BChE* gene; (**D**) *ACHE* gene; (**E**) *GSTM1* gene; and (**F**) *GSTT1* gene.

Variation in glutathione level according to genotypes (Figure 2A) showed statistical signficant differences between genotypes and serum GSH levels in the Pakistani exposed subjects compared to the non-OP-exposed subjects, but the result was not significant in the Cameroon exposed groups.

Difference in allele and genotype frequencies of CAT rs7943316 among exposed subjects and non-OP-exposed of both countries were statistically significant. Significant association of CAT with risk of OPs toxicity was revealed in Pakistani (DM: OR = 0.394, allele) and in Cameroonian (OR = 0.422 for DM, only) exposed group (Table 4). There are significant differences in catalase levels according to CAT SNP genotypes in OP-exposed subjects compared to non-OP-exposed in the Pakistani and Cameroonian populations (Figure 2B).

In the case of BCHE (SNP rs3495), significant association of BCHE risk allele with susceptibility to OPs toxicity was obseved in Pakistani OP-exposed subjects in all inhertance models (Table 4), while significant association was noted in DM and allelic modals for Cameroonian exposed subjects (Table 4).

Regarding AChE SNP rs2571598 allele and genotype frequencies between exposed subjects and non-OP-exposed were significantly different in Pakistani population. On the other hand, only genotypic frequencies’ differences were statistically significant in the Cameroonian population (Table 4). A significant association between ACHE SNP rs2571598 and susceptibility to OPs toxicity was identified in Pakistani exposed subjects in all inheritance models, whereas in Cameroonian exposed group significant association was noted only in DM inhertance model (Table 4).

SIRT1(rs10823108) revealed no substantial differences in allele frequencies and genotypes between OP-exposed and non exposed in both countries. Consequently, no association between these SNPs and risk to OPs exposure toxicity has been identified among Pakistanis and Cameroonians. Comparison of OP-exposed and non exposed with GSTT1 and GSTM1 null loci (Table 3) showed statistically significant differences only in the Cameroonian population (OR = 0.449 and 95% CI = 0.252–0.800 for GSTM1; OR = 0.459 and CI = 0.251–0.839 for GSTT1).

**Figure 3:**
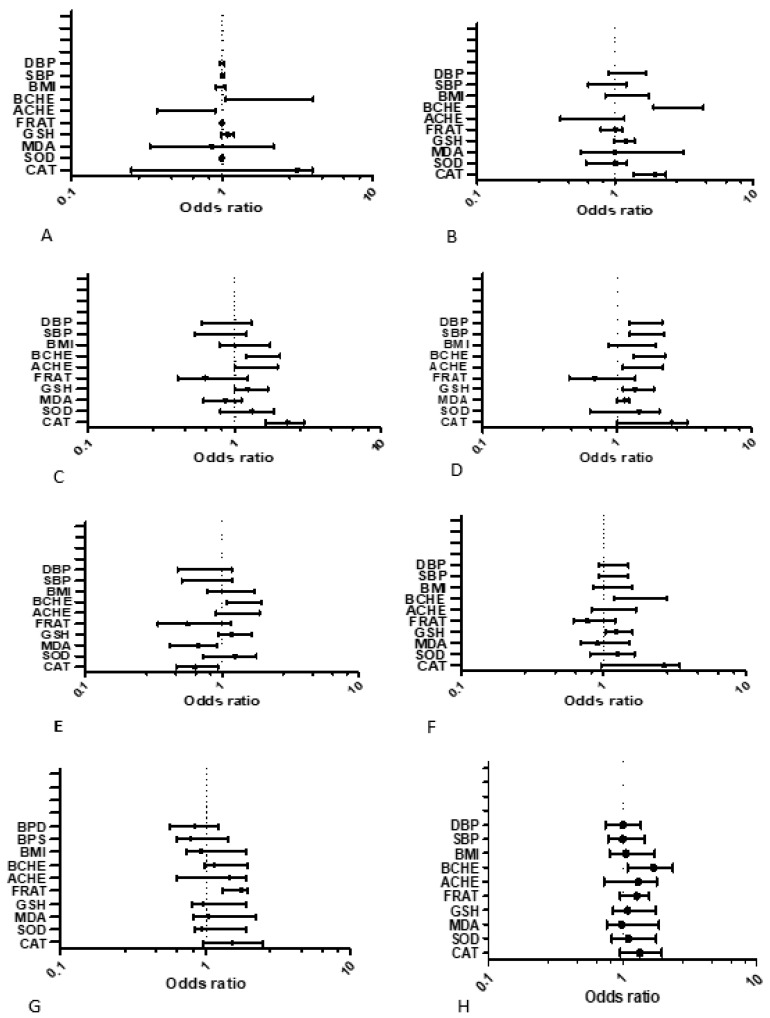
Multiple Logistic regression (MLR) analysis of genetic polymorphisms (SNPs) in dominant model according to Biochemical Variables of OPs-exposed population: The model was adjusted for each SNPs (0: major homozygote; 1: heterozygote + minor homozygote). The results were significant if *p* ≤ 0.05. (**A**) MLR for *GSTP1* gene of Pakistan; (**B**) MLR for *GSTP1* gene of Cameroon; (**C**) MLR for catalase gene of Pakistan; (**D**) MLR for gene of exposed Cameroon; (**E**) MLR for *BCHE* gene of Pakistan; (**F**) MLR for *BCHE* gene of Cameroon; (**G**) MLR for *ACHE* gene of Pakistan; and (**H**) MLR for *ACHE* gene of Cameroon.

Logistic regression analysis of different gene SNPs (rs1695, rs7943316, rs3495, and 2571598) in dominant model according to studied biochemical parameters (Figure 3A–H) showed statistically significant differences for cholinergic enzymes (AChE and BChE) and antioxidant enzymes (CAT, SOD, and MDA) in OPs-exposed individuals of both countries. *ACHE* SNPs rs2571598 was positively associated with BMI (*p* = 0.04; OR = 0.92; CI = 0.74–1.91) in Pakistani population (Figure 3G). It was also associated with FRAP levels (*p* = 0.04; OR = 1.30; CI = 0.96–1.58) in the Cameroonian population (Figure 3H).

## 3. Discussion

Organophosphorus compounds makeup about 80% of the total insecticides/pesticides consumption across the world. Globally, thousands of peoples die each year due to pesticide poisoning [1]. Intoxication and maladies due to acute and chronic exposure in populations is regularly reported in literature [29,30,31,32,33]. Mixture of OPs even at low non-lethal dose may pose enhanced threat because of the possibility of synergistic effect between two OPs [34,35]. Reduction of AChE activity is an essential biological marker for OPs exposure [6,36,37]. A significant decrease in cholinergic enzymes (AChE and BChE) in OP-exposed groups were noted (Table 2) in the study, indicating the OPs exposure in research participants. The OPs exposure was further confirmed by presence of empty pesticides containers in the area, in addition to the verbal inquiry from pesticides applicator in the region. Blood pesticides residues were determined in the plasma which showed the presence of three types of OPs (malathion, parathion, and chlorpyrifos) in theOP-exposed groups (Figure 1).

Our results indicate that chronic exposure of OPs pesticides can lead to an imbalance in antioxidant enzymes. Oxidative stress has been reported as one of the toxic manifestation of organophoshates and a co-morbidity and mortality factor in OP poisoning [8]. However, controversialresults also exists which is attributed to the structurally and functionally diversified organophosphates [8]. However, reports are mainly based on in vitro and animal models studies on organ toxicity. [38,39,40,41]. Secondly, most of the studies were related with mild toxic OPs like malathion [42,43], whereas extremely toxic OP, for instance, paraoxon or mixtures of Ops, may exhibit different toxicological profiles, although they have a common mechanism of poisoning that is inhibition of AChE [10,11]. In the present study, a mixture of OPs malathion+chlorpyrifos+/parathion) residues were found in chronically exposed subjects and exhibited enzymatic anti-oxidants irregularities. Damage made by oxidative stress mainly happens through the production of ROS [44] and one of the ROS deactivating pathways is mediated by catalase.

A significantly lower activity of CAT and SOD levels in OP-exposed peoples compared to non-OP-exposed subjects was observed in both populations groups. GSH was found to be decreased only in Pakistani exposed group. A decrease in activity of these enzymes would favor the accumulation of free oxygen radicals in erythrocytes and other cells, leading to tissue damage which could be significantly detrimental when pesticides exposure persists over several years and may ultimately be responsible for multipletissue and organ damages (neurological, metabolic, hepatic, and renal) [45,46,47]. We also observed an increase in MDA and FRAP in OP-exposed individuals. A higher level of MDA and FRAP, could suggest an increase in lipid peroxidation in exposed individuals. Our observation is consistent with previous studies where a reduction in AChE activity was correlated with a decrease in antioxidant enzymes and an increase in lipid peroxidation after sub-chronic and chronic OPs exposure [48,49].

It is evident from the literature thatpresence of certain SNPs in detoxyfying/antioxidant pathway genes helps in predicting the individual’s susceptibility to pesticide induced toxicity. [50,51]. Moreover, genotypes determine the modulations of proteins and enzymes involved in DNA metabolism, detoxification, and repair systems, influencing heterogeneity of responses to pesticides and their metabolism [52]. SNPs analysis on selected cholinergic and antioxidant genes have revealed significant association of *ACHE* rs2571598, *BCHE* (rs3495), and *CAT* (rs7943316) with lower levels of their respective enzyme activity. Allelic variants of *CAT* may exhibit deleterious effect and may result in lower expression of catalase enzyme activity [53], hence implicating it in the risk of toxicity or sensitivity due to chronic OPs exposure.

GSHs are a group of enzymes (GSTP1, GSTT1, and GSTM1) which deactivate free oxygen radicals rendering them unable to interact with other proteins and enzymes having key roles in cellular function [54,55]. Although, there was no significant correlation between GSTP1 (rs1695) genotypes and GSH levels in both OP-exposed subjects of both populations in this study. The results of null variants of GSTT1 in Cameroonian and Pakistani populations appeared also not to be correlated with low levels of GSH. There could perhaps be an adaptive response of erythrocytes to protect themselves against damaging oxidative stress and to protect the key biochemical processes and pathways in OP-exposed individuals.

In our study, a multivariate logistic analysis of dominant model of each SNP according to the different biochemical parameters was carried out, in osrder to assess the contribution of these SNPs of interest on toxicological process as well as on other variables such as than BMI andhypertension. We identified that in general, there are relevant overlapping effects, for example, cholinergic enzymes (AChE and BChE) and antioxidant enzymes (catalase, SOD and MDA) were significantly associated with each of the SNPs studied. This association between the SNPs and biochemical variables implies that, in our study populations (Cameroonian and Pakistani) the presence of the mutant allele is positively correlated with considerabe increase in risk of homeostatic dysfunction of these cholinergic and antioxidant enzymes The ACHE SNP rs2571598 was also positively associated with BMI in Pakistani population. Additionally, the same ACHE SNPs was associated with FRAP levels in Cameroonian population. Although, these gene SNPs have been associated in several other studies with different diseases [55,56,57,58], our work, with respect to toxicogenetics, is the first of its kind to link the influence of these five studied SNPs with cholinergic, and oxidative profiles in chronic OP-exposed groups.

## 4. Methodology

### 4.1. Study Design and Recruitment of Study Subjects

This study was cross-sectional and subjects from an intensive agricultural area, making use of heavy pesticides, of Pakistan and Cameroon were recruited to participate in the study. The study participants were recruited from Mora, Figuil, Njobe, and Sa’a in Cameroon and, from Depalpur and Multan in Pakistan. These are cotton growing agriculture areas where organophosphates are mainly applied. Ethical review board of COMSATS University Islamabad, Pakistan (CIIT/BIO/ERB/19/90) and Cameroon National Ethical Committee (#488/CE/CNERSH) approved the study. The study confirms to tenets of the Helsinki Declaration for human subjects in experiments. All the study subjects were told about the purpose of the study and written consent was obtained before sample collection. The age of study subjects was in the range of 16–60 years, had lived in the OPs sprayed agriculture areas for at least 6 months and exposed to pesticides directly or indirectly.

All the subjects disclosing that he/she has used pesticides other than OPs; people with diabetes, neurological disorders, liver dysfunction, cancer or any other chronic condition; and those whose gas chromatography coupled with high-resolution mass spectrometry (GC–MS) results revealed presence of other pesticide residues than OPs were excluded from the study. Since all the inhabitants in the understudied agriculture areas were directly or indirectly exposed to pesticides, unexposed samples were collected from non-agriculture areas.

Questionnaires were provided to collect demographic characteristics and confounding factors such as age, gender, height, weight, tobacco, work practices, exposure history, use of protective equipments, length of time doing present work activity, and proximity of home to agricultural fields were recorded. The sample size was calculated using the Lorenz formula for a cross-sectional study. According to pancreatic disease prevalence in respective populations (7.9% in Pakistan and 7.1% in sub-Saharan Africa), the minimum size for each site required to have enough statisitcal power (with 10% increase to keep the power in case of selection bias) was 300 for each country. After screening and exclusion, a total of 637 participants were selected to participate in the study where 200 were exposed and 125 unexposed from Cameroon, and 200 exposed and 112 unexposed from Pakistan.

Sampling method was non-probabilistic. Blood samples were drwan once from all participants using aseptic venipuncture and transported to the lab on ice. The collected blood was divided into two tubes for subsequent analysis for plasma and serum fractions, using EDTA coated and plain glass tubes respectively. The blood was centrifuged at 3500 rpm for 10 min at 25 °C. Plasma and Serum thus obtained were then stored at −80 °C till further analysis. The samples were analyzed at the Institute of Medical Research and Medical Plant studies(IMPM)laboratory, Yaounde, Cameroon and Department of Biosciences laboratories at COMSATS University Islamabad, Pakistan.

### 4.2. Biochemical Analysis

#### 4.2.1. Exposure Measures

Hernández et al. and Nurulain et al. [7,59] have previously shown that cholinesterase activities particularly AChE can be used as a marker to measure acute and/or chronic exposure to OPs pesticides. RBC-acetylcholinesterase (RBC-AChE) and butyrylcholinesterase (BChE) activities were determined according to Worek et al. [60] using Specord 50 plus spectrophotometer (Number; 233H1280C, Analytic Jena, Jena, Germany). RBC-AChE was measured from whole blood and plasma was used for BChE. Blood and plasma dilutions were prepared according to the method described by Worek et al. [60]. Spectrophotometric measurements were taken at 436 nm (ε = 10.6 × 10^3^ M^−1^ cm^−1^) at 37 °C for AChE and BChE. For hemoglobin, absorption was noted at 546 nm (ε = 10.8 × 10^3^ M^−1^ cm^−1^).

To confirm the presence of pesticides in blood, gas chromatography coupled with high-resolution mass spectrometry (GC–MS, System 5975C Agilent, Santa Clara, CA, USA) according to Pérez et al. [61] protocol with minor modifications was employed.

#### 4.2.2. Measurement of Oxidative Stress Parameters

Malondialdehyde (MDA) was determined in a colorimetric assay using thiobarbituric acid as described by Wilbur. In fact, carbonyl compounds such as malondialdehyde from lipid oxidation and the breakdown of fatty acid hydroperoxides react with thiobarbituric acid (TBA) to give pink chromophores. The absorbance of that pink chromophores was read at 532 nm (ε = 1.53 × 10^5^ M^−1^ cm^−1^) and is proportional to the concentration of MDA. The result is expressed in nmol·MDA/mg protein [62].

The reduced glutathione (GSH) was determined using the method previously described by Ellman (1959) with minor modification [63]. The reaction consists of coupling 2,2-dithio-5,5′-dibenzoic acid (DTNB) with thiol (SH) groups of glutathione, to form a colored complex (thionitrobenzoic acid or TNB). TNB at alkaline pH (8–9) absorbance at 412 nm (ε = 13600/M·cm.) The intensity of the staining was proportional to the concentration of the SH groups and the result was expressed in ug/mL.

Measurement of superoxide dismutase (SOD) was done spectrophotometrically according to Misra and Fridovich methods [64]. The auto-oxidation of epinephrine in presence of EDTA is inhibited by SOD at pH 10.2. The principle of the assay was based on competition between the oxidation reaction of epinephrine (4.5 mM) by superoxide anion (O_2_^−^). The absorbance of the reaction was read after 30 s and 150 s at 480 nm (4020/M.cm). For activity calculation 1 unit (U) of SOD is that the amount of SOD needed to cause 50% inhibition of the oxidation of adrenaline to adrenochrome for one min

The ferric reducing ability of plasma (FRAP) as a measure of antioxidant power was measured with Benzie and Strain method with few modifications [65]. The method measuring the ability of serum to reduce iron in an acidic environment (pH about 3.6). An intense blue color was formed when ferric tripyridyltriazine complex (TPTZ) was reduced to ferric tripyridyltriazine and absorbance was measured at 593 nm and the result was expressed in µM.

Catalase was measured by evaluating the transformation of dichromate-acetate as described by Boutin et al. [66]. The method is based on the fact that dichromate in acetic acid is reduced to chromic acetate when heated in the presence of H_2_O_2_, with the formation of perchromic acid as an unstable intermediate. The chromic acetate thus produced is measured at 570 nm. Catalase activity was calculated in µmol/min/mg protein and was expressed as U/mg protein.

Total proteins were measured according to Biuret method: in basic medium, sodium tartrate of sodium and potassium form with cupric ions a soluble complex. Addition of a protein displaces the complex of copper with tartrate to form another copper-protein complex. Absorption was read at 540 nm and staining intensity was proportional to protein concentration in the medium and expressed in mg [67]. All the measurements were done using Kenza spectrophotometer (Number; 450 TX, Biolabo, France).

### 4.3. Primer Designing and Chemicals

Primers designing for the SNPs of selected genes was performed with the Primer 3 version 0.4.0 (http://bioinfo.ut.ee/primer3-0.4.0/primer3/). NCBI Blast software (https://www.ncbi./Nlm.nih.gov/tools/primer-blast/) and PCR-in silico using the University of California Santa Cruz (UCSC) genome browser (https://genome.ucsc.edu./cgi-bin/hgpcr) was used to confirm the specificity of the primers All primers were obtained from Macrogen Inc. (Rockville, MD, USA). Primer sequences of all SNPs are given in the Supplementary Data File (Table S1). All the chemicals were obtained from Thermo Fisher Scientific (Waltham, MA, USA) and Sigma-Aldrich (St Louis, MO, USA).

### 4.4. Genomic DNA Extraction and SNP Genotyping

Genomic DNA was extracted by an earlier described method by Lahiri and Nurnberger [68] with slight modifications. Precipitation of DNA was achieved with pure ethanol and stored in TE buffer at −20 °C until further use. Genotyping was carried out by PCR-RFLP method and PCR products were incubated at 37 °C for 14–16 h with respective restriction enzymes (RE) as follows:

- For SIRT1(rs10823108), RE Hpy188I (Catalog # ER0761, Thermo Fisher Scientific) cleaves the PCR product into 2 fragments of 261 bp and 134 bp in presence of major A allele while those PCR products having minor G allele remains intact (band size of 395 bp).
- For GSTP1 (rs1695), RE BsmAI (Catalog # ER0761, Thermo Fisher Scientific) cleaves the PCR product into 2 fragments of 201 bp and 148 bp in presence of major G allele while those PCR products having minor A allele remains intact (band size of 349 bp).
- For CAT (rs7943316), RE MlyI (Catalog # ER0761, Thermo Fisher Scientific) cleaves the PCR product into 2 fragments of 209 bp and 158bp in presence of major A allele while those PCR products having minor T allele remains intact (band size of 367 bp).
- For ACHE (rs2571598), RE Bsu36I (Catalog # ER0761, Thermo Fisher Scientific) cleaves the PCR product in 2 fragments of 210 bp and 125 bp in presence of major C allele while those PCR products having minor T allele remains intact (band size of 335 bp). RE BanII (Catalog # ER0761, Thermo Fisher Scientific) cleaves the PCR product into 2 fragments of 209 bp and 126 bp in presence of major G allele while those PCR products having minor T allele with band size of 335 bp remains intact.
- For BCHE (rs3495), RE NSP1(XceI) (Thermo Fisher Scientific Catalog# ER1471). The product bearing A allele at rs3495 gave two fragments of 225 bp and 144 bp length, while the G allele bearing products remain intact and observed band length was 369 bp.
- For performed analyses of GSTM1 and GSTT1 genes deletion, we used co-amplification of the gene HBB as an internal non-OP-exposed.

### 4.5. Statistical Analysis

Stata 15, R 3.2.0, and Graphpad Prism 7.0 (Graphpad Software Inc., La Jolla, CA, USA) softwares were used for statistical analysis of the data. The normality of the distribution of the variables was established using the Shapiro–Wilk test. The χ2 test was used to verify the agreement of the observed genotype frequencies with those expected according to the Hardy–Weinberg equilibrium. ANOVA test, and post-hoc tests were performed where necessary. A logistical regression model was used to establish a relationship between the explained variables (DM, Genotype) and the explanatory variables (Biochemical Parameters). Odds ratios (ORs) with 95% confidence intervals (95% Cis) were calculated. Statistical significance was set at *p* ≤ 0.05.

## 5. Limitations

A possible limitation of the present study could be that OPs, although they are used extensively in chosen regions in Pakistan and Cameroon, is the presence of other potential co-influencing factors, such as heavy metals, e.g., arsenic or lead, have not been considered. Another limitation of our work is the lack of measurement of certain specific mitochondrial parameters of oxidative stress such as 8-oxoguanine (8-oxoG) or Myeloperoxidase (MPO) that better explain the effect of oxidative stress of DNA damage. For instance, the measurement of 8-oxoG can be very important under OPs exposure condition because the accumulation of 8-oxoG, can cause mitochondrial dysfunction thus, increasing the chance of genetic problems.

## 6. Conclusions

The study concludes that long term exposure of mixture of OPs significantly disbalance the antioxidants in human subjects. The SNPS were associated with investigated oxidative stress parameters and cholinergic enzymes in the population of both groups. the Toxicogenetics screening revealed a substantial association with catalase SNP CAT (rs7943316), GSTP1 (rs1695), and Gene GSTM1 and OPs exposure in both Cameroon and Pakistani groups. Cholinergic enzymes SNPs ACHE (rs2571598), and BCHE (rs3495) also showed a significant association with toxic effects of OPs exposure. Further studies with different ethnic groups and other antioxidant pathway gene SNPs are suggested in order to understand mechanism under pining gene-pesticide interaction and related health outcomes in vulnerable population groups.

## Figures and Tables

**Table 1:** Sociodemographic characteristics of study participants.

Groups	Cameroon			Pakistan		
	Unexposed *n* (%)	Exposed *n* (%)	*p*-Value	Unexposed *n* (%)	Exposed *n* (%)	*p*-Value
Age-frequency						
16–30	80 (64)	109 (54.5)	0.158	38 (33.9)	115 (57.5)	0.146
31–45	25 (20)	71 (35.5)		49 (43.8)	60 (30)	
46–61	20 (16)	20 (10)		25 (22.3)	25 (12.5)	
Gender-frequency						
Female	63 (50.4)	100 (50)	0.602	46 (41.1)	52 (26)	<0.05
Male	62 (49.6)	100 (50)		66 (58.9)	148 (74)	
BMI-frequency						
Underweight	8 (6.4)	9 (4.5)	<0.001	4 (3.6)	11 (5.5)	<0.001
Normal range	81 (64.8)	32 (16)		63 (56.2)	50 (25)	
Overweight	13 (10.4)	91 (45.5)		38 (33.9)	89 (44.5)	
Obese	23 (18.4)	68 (34)		7 (6.2)	50 (25)	
SBP-frequency						
Hypotension	15 (12)	3 (1.5)	<0.001	8 (7.1)	11 (5.5)	<0.001
Normal range	90 (72)	80 (40)		88 (78.6)	77 (38.5)	
Hypertension	20 (16)	117 (58.5)		16 (14.3)	112 (56)	
DBP-frequency						
Hypotension	9 (7.2)	8 (4)	<0.001	9 (8.0)	15 (7.5)	<0.001
Normal range	86 (68.8)	60 (30)		91 (81.2)	72 (36)	
Hypertension	30 (24)	132 (66)		12 (10.7)	113 (56.5)	

*n* = number of patients, SBP = systolic blood pressure, DBP = diastolic blood pressure, BMI = Body mass index.

**Table 2:** Status of cholinergic enzymes in unexposed and OP-exposed groups.

Groups	Cameroon		Pakistan	
	Unexposed (*n* = 125) Mean ± SD	Exposed (*n* = 200) Mean ± SD	Unexposed (*n* = 112) Mean ± SD	Exposed (*n* = 200) Mean ± SD
AChE mU/µmol Hb	0.21 ± 0.09 ^c^	0.12 ± 0.05 *^,a^	0.33 ± 0.07 ^d^	0.19 ± 0.06 *^,b^
BChE µmol/l/min	0.021 ± 0.009 ^b,c^	0.019 ± 0.010 *^,a^	0.032 ± 0.006 ^c^	0.022 ± 0.009 *^,b^

SD = Standard deviation, * *p* ≤0.05; ^a, b, c, d^ = Significance degree for Tukey Post-hoc test (Anova-one way).

**Table 3:** Status of antioxidant enzymes in unexposed and OP-exposed groups.

Groups	Cameroon		Pakistan	
	Unexposed (*n* = 125) Mean ± SD	Exposed (*n* = 200) Mean ± SD	Unexposed (*n* = 112) Mean ± SD	Exposed (*n* = 200) Mean ± SD
Catalase U/mg protein	0.714 ± 0.607 ^d^	0.301 ± 0.201 *^,b^	0.513 ± 0.240 ^c^	0.223 ± 0.110 *^,a^
SOD U/mg protein	51.88 ± 32.78 ^b^	41.07 ± 13.09 *^,a^	42.62 ± 19.35 ^a,b^	33.54 ± 16.33 *^,a^
MDA nmol MDA/mg protein	0.89 ± 0.41 ^a^	1.95 ± 0.85 *^c^	0.99 ± 0.31 ^a^	1.22 ± 0.37 *^,b^
GSH μg/mL	21.81 ± 4.43 ^a^	21.26 ± 6.61 ^a^	29.55 ± 5.18 ^b^	21.77 ± 4.10 *^,a^
FRAP μM	122.31 ± 104.85 ^a^	419.50 ± 238.81 *^,d^	252.54 ± 56.07 ^b^	351.65 ± 97.70 *^,c^

* *p* ≤ 0.05; ^a, b, c, d^ = Significance degree for Tukey Post-hoc test (Anova-one way).

**Table 4:** Genotypes and Alleles frequencies in unexposed and OP-exposed groups.

Genotype		Cameroon				Pakistan			
		Unexposed *n* (%)	Exposed *n* (%)	OR (95% CI)	χ2 (*p*-Value)	Unexposed *n* (%)	Exposed *n* (%)	OR (95% CI)	χ2 (*p*-Value)
*GSTP1*	AA	70 (56)	84 (42)		6.167 (0.045)	34 (30.36)	47 (23.5%)		1.810 (0.404)
	AG	34 (27.2)	75 (37.5)			57 (50.89)	114 (57)		
	GG	21 (16.8)	41 (20.5)			21 (18.75)	39 (19.5)		
	DM AG + GG vs. AA			0.569(0.362–0.893)	6.047 (0.013)			0.704 (0.419–1.184)	1.756 (0.185)
	RM AG + AA vs. GG			1.240 (0.692–2.220)	0.526 (0.468)			1.050 (0.582–1.893)	0.026 (0.871)
	A	174 (69.6)	242 (60.5)	0.666 (0.476–0.932)	5.641 (0.017)	125 (55.80)	208 (52)	0.858 (0.617–1.192)	0.834 (0.360)
	G	76 (30.4)	158 (39.5)			99 (44.20	192 (48)		
*CAT*	AA	68 (54.4)	67 (33.5)		14.05 (0.009)	67 (59.82)	74 (37)		15.80 (0.001)
	AT	48 (38.4	115 (57.5)			30 (26.78)	93 (46.5)		
	TT	9 (7.2)	18 (9)			15 (13.40)	33 (16.5)		
	DM AT + TT vs. AA			0.422 (0.267–0.667)	13.84 (0.002)			0.394 (0.245–0.634)	15.10 (0.001)
	RM AT + AA vs. TT			1.275 (0.553–2.934)	0.327 (0.567)			1.278 (0.660–2.472)	(0.532) 0.465
	A	184 (73.6)	249 (62.25)	0.554 (0.388–0.792)	8.912 (0.002)	164 (73.21)	241 (60.25)	0.591 (0.418–0.836)	10.59 (0.001)
	T	66 (26.4)	151 (37.75)			60 (26.79)	159 (39.5)		
*BCHE*	GG	26 (20.8)	33 (16.5)		34.97 (0.000)	56 (50)	70 (35)		8.695 (0.01)
	GA	70 (56)	56 (28)			35 (31.25)	66 (33)		
	AA	29 (23.2)	111 (55.5)			21 (18.75)	64 (32)		
	DM GA + AA vs. GG			1.329 (0.750–2.353)	0.957 (0.327)			1.857 (1.160–2.974)	6.710 (0.01)
	RM GA + GG vs. AA			0.247 (0.149–0.408)	31.61 (0.000)			0.490 (1.979–16.58)	12.73 (0.001)
	G	122 (48.8)	122 (30.5)	0.460 (0.332–0.638)	21.97 0.000	147 (65.63)	206 (51.5)	0.556 (0.280–0.858)	6.359 (0.01)
	A	128 (51.2)	278 (69.5)			77 (34.37)	194 (48.4)		
*ACHE*	GG	85 (68)	110 (55)		9.843 (0.007)	70 (62.5)	78 (39)		21.14 (0.001)
	GT	23 (18.4)	69 (34.5)			38 (33.93)	87 (43.5)		
	TT	17 (13.6)	21 (10.5)			4 (3.57)	35 (17.5)		
	DM GT + TT vs. GG			0.575 (0.360–0.918)	5.417 (0.019)			0.383 (0.238–0.617)	15.90 (0.001)
	RM GT + GG vs. TT			0.745 (0.376–1.475)	0.715 (0.397)			5.72 (1.979–16.58)	12.73 (0.001)
	G	193 (77.2)	289 (72.25)	0.768 (0.532–1.111)	1.967 0.160	178 (79.46)	243 (60.75)	0.400 (0.273–0.585)	22.91 (0.001)
	T	57 (22.8)	111 (27.75)			46 (20.54)	157 (39.25)		
*SIRT1*	GG	28 (22.4)	39 (19.5)		2.5 (0.286)	45 (40.18)	81 (40.5)		0.23 (0.891)
	GA	82 (65.6)	124 (62)			44 (39.29)	74 (37)		
	AA	15 (12)	37 (18.5)			23 (20.53)	45 (22.5)		
	DM GA + AA.Vs GG			0.839 (0.485–1.45)	0.395 (0.529)			1.013 0.632–1.624	0.003 (0.957)
	RM GA + GG vs. AA			1.665 (0.871–3.179)	2.41 (0.119)			1.123 (0.637–1.979)	0.162 (0.686)
	G	138 (55.2)	202 (50.5)	0.828 (0.602–1.137)	1.362 (0.243)	134 (59.82)	236 (59)	0.966 (0.692–1.349)	0.040 (0.841)
	A	112 (44.8)	198 (49.5)			90 (40.18)	164 (41)		
*GSTM1*	Present (+)	106 (84.8)	143 (71.5)	0.449 (0.252–0.800)	7.595 (0.005)	96 (85.71	175 (87.5)	1.167 (0.593–2.29)2	0.200 (0.654)
	Absent (−)	19 (15.2)	57 (28.5)			16 (14.29)	25 (12.5)		
*GSTT1*	Present (+)	108 (86.4)	149 (74.5)	0.459 (0.251–0.839)	6.584 0.010	94 (83.93)	172 (86)	1.176 (0.618–2.239)	0.245 (0.620)
	Absent (−)	17 (13.6)	51 (25.5)			18 (16.07)	28 (14)		

## Supplementary Materials

The following are available online at https://www.mdpi.com/1422-0067/21/17/6432/s1, Table S1: Primer sequences of study SNPs.
