# Supplementary material for: Oxidative Stress and Analysis of Selected SNPs of ACHE (rs 2571598), BCHE (rs 3495), CAT (rs 7943316), SIRT1 (rs 10823108), GSTP1 (rs 1695), and Gene GSTM1, GSTT1 in Chronic Organophosphates Exposed Groups from Cameroon and Pakistan
Source: Int J Mol Sci. 2020 Sep 3;21(17):6432. doi: 10.3390/ijms21176432 (PMC7503738; doi:10.3390/ijms21176432)
Supplement: Supplementary file 1 [file ijms-21-06432-s001.pdf]

Table S1: Primer sequences of study SNPs

**Primer's ID**	**Primer's sequences**	**Product size**
ACHE rs2571598<br>ACHE-F<br>ACHE-R	 5'CTCACCTTCCCAAGTAGCTG3'<br>5'CAGGATAGAACAGCCAGAGAG3'	 335bp
BCHE rs3495<br>BCHE-F<br>BCHE-R	 5´CACTAGCAAGAAAGAAAGTTGTGTG3´<br>5´AATACACGTGACTAAAAGCAGAGC-3´	 369bp
SIRT1 rs10823108<br>SIRT1-F<br>SIRT1-R	 5'CATGCCCTGCTGTTTCTTTG3'<br>5' GGACTACTAAAGGCTGGAATCC3'	 395bp
CAT rs7943316<br>CAT-F<br>CAT-R	 5'TGGGTATCTCCGGTCTTCAG3'<br>5'CGCTTTCTAAACGGACCTTC3'	 367bp
GSTP1 rs1695<br>GSTP1-F<br>GSTP1-R	 5'CAGTGACTGTGTGTTGATCAGG3'<br>5'ATAAGGGTGCAGGTTGTGTC3'	 349bp
GSTM1<br>GSTM1-F<br>GSTM1-R	 5GAACTCCCTGAAAAGCTAAAGC'3'<br>5'GTTGGGCTCAAATATACGGTGG3'	 219bp
GSTT1<br>GSTT1-F<br>GSTT1-R	 5'TTCCTTACTGGTCCTCACATCTC3'<br>5'GCATCAGCTTCTGCTTTATGG3'	 433bp
HBB<br>HBB-F<br>HBB-R	 5'GAAGAGCCAAGGACAGGTAC3'<br>5'GGAAAATAGACCAATAGGCAG 3'	 408bp
